# Editorial: The role of biomechanics in anterior cruciate ligament injuries prevention

**DOI:** 10.3389/fspor.2023.1134969

**Published:** 2023-03-09

**Authors:** David J. Saxby, Danilo S. Catelli, David G. Lloyd, Zimi Sawacha

**Affiliations:** ^1^ Griffith Centre of Biomedical and Rehabilitation Engineering (GCORE), Menzies Health Institute Queensland, Griffith University, Gold Coast, QLD, Australia; ^2^ Department of Movement Sciences, Faculty of Movement and Rehabilitation Sciences, KU Leuven, Leuven, Belgium; ^3^ Department of Information Engineering, University of Padua, Padua, Veneto, Italy; ^4^ Department of Medicine, University of Padua, Padua, Veneto, Italy

**Keywords:** anterior cruciate ligament (ACL), biomechanics, injury prevention, rehabilitation, ACL reconstruction

**Editorial on the Research Topic** The role of biomechanics in anterior cruciate ligament injuries prevention

It is with optimism and pleasure we write this editorial for Frontiers in Sports and Active Living, Injury Prevention and Rehabilitation. We acknowledge the work of our colleagues made in response to the well-documented problem of anterior cruciate ligament (ACL) injuries in our community and the numerous issues surrounding their rehabilitation. We note the increasing rates of ACL globally (1), the peculiar rise among adolescents, and the marked risk for adolescent/young adult females (2, 3). The increasing rates of ACL injury indicate that whatever injury prevention programs that have been deployed to date have been unsuccessful in achieving their aims.

Unfortunately, re-injury rates following ACL reconstruction have been reported to be as high as 24% (4, 5). Moreover, roughly only 65% of ACL-injured patients return to pre-injury levels of sport while 55% return to competitive sport (6). We are dealing with a confluence of issues: injury rates have been rising precipitously, risk reduction programs to date have had limited effects, and secondary injury as well as long-term health impairment are common following surgical reconstruction. Clearly, there is an urgent need for improved programs for risk reduction and enhanced rehabilitation.

With all humility, we remind the biomechanics and clinical communities that we *must* do something different to our current approach if we are to expect different (and hopefully improved) results. In this special issue, our colleagues touch on potentially viable in-field monitoring methods, female-specific biomechanics, post-ACL reconstruction muscle morphology, and neuromuscular features following ACL reconstruction. In our commentary, we highlight the positive contributions of this work and point a way forward for the field.

First, a reminder to readers that ACL injuries do not occur in scientific laboratories, but during participation in sports and recreation. If we are to study the ACL injury event and its associated biomechanics, we require technology that can be deployed in ecologically valid contexts (e.g., sports fields, practice courts). Such technology must be capable of operating unobtrusively in the field and adopted widely within the community to capture multiple injury events.

Undoubtedly, when using field-friendly technology there will be a loss of data quality compared to laboratory instruments, both due to technical considerations of measurement but also due to limited knowledge from users. To what degree this data quality loss might have on the interpretation of results is unclear, and likely application specific. To this end, Ulman et al. and colleagues assessed the agreement between a subjective scoring of low-dimensional kinematic data (i.e., 2D videography) with 3D kinematics acquired using laboratory-based instruments. Overall, they found moderate agreement between methods, which is encouraging as 2D videography is currently a readily accepted technology in sports and rehabilitation settings from community to professional levels. However, it is critical to note this study examined movements performed in the laboratory. The agreement between subjective assessment of 2D videography and 3D kinematics may not be extensible to on-field movements as they are likely more complex, performed faster, and may have serious contextual challenges (e.g., lighting, obstruction of view, camera positioning). As it relates to the injury itself, it is also worth noting that kinematics themselves are not the mechanical causes of tissue injuries such as ACL rupture. Rather, the body, inertial, joint, and muscle forces are, collectively, what creates human kinematics, and their complex interaction is what results in soft tissue loads, subsequent strains, and eventual injury. A 2D screening tool cannot assess tissue loading on its own, but the move towards field-friend low-dimensional technology is, in our opinion, the correct direction.

Although field-friendly kinematic assessment is a promising early step, several additional technologies must be coupled to videography analysis to gain insight into tissue mechanics. In this respect, **Spolaor and Ciniglio et al**. applied an on-field screening that provides information both at the level of joint kinematics and kinetics, based on a combined 3D video and plantar pressure analysis similar to what proposed in Guiotto et al. (7), by the same authors. An elite female soccer team was assessed, before and after the administration of proprioceptive stimuli at four different time points, while performing a series of four side cuts. The most common biomechanical variables, generally associated with the risk of ACL injury (i.e., knee flexion angle, knee flexion moment, knee valgus moment) were evaluated (8, 9). The authors hypothesized ACL injury prevention could be enhanced by improving athletes' motor control through the application of stimuli to the proprioceptive system. The supposition being these stimuli would result in changes to the biomechanics of “high-risk” movements (i.e., sidestep cutting maneuver) linked to a noncontact ACL injury (10), which has also been observed during fatigue protocol (11). Overall, the biomechanics world is moving towards in-field technologies capable of robustly monitoring tissue loading in valid contexts.

Clearly, the field of biomechanics is making deep inroads toward field-capable tools for tissue monitoring. When realized, this will be an outstanding technological achievement. However, as noted in our introductory remarks, young females are particularly at risk of ACL injury compared to their male counterparts. The explanations for why remain obscure, even when analysed in the laboratory setting. A host of reasons may explain these sex-based discrepancies in ACL injury rates relating to anatomy, movement and muscle coordination, hormonal changes, and physical conditioning. However, ultimately the ACL is broken due to excessive strains on the tissue caused by applied (tensile) stresses, which can now be computed using mathematical simplifications (12).

One particularly potent biomechanical variable shown to strain the cadaveric knee is valgus knee moments (13, 14). In the study by Bill et al. it is reported that athletes with large knee valgus moments also demonstrate large vertical center of mass excursion (i.e., dynamic range) and knee valgus posture when performing provocative motor tasks that likely challenge knee stability. Although knee valgus moment is well acknowledged as both a statistical and mechanistic risk factor for ACL injury, this study notes the coupling between body dynamics (i.e., centre of mass vertical excursions) and knee valgus posture in those with large valgus moments. The result may be counterintuitive for many, as a greater centre of mass excursion could be achieved by creating limb compliance which could lower peak reaction forces and joint moments. However, trunk flexion (Bill et al. report only lateral flexion and rotation) might accommodate the increased centre of mass excursion with minimal changes to lower limb compliance. The authors assume the coupling between the centre of mass excursion velocity and knee valgus posture to be deeply rooted in the individual, owing to their anatomy and years of motor practice. Therefore, modulating the vertical centre of mass kinematics and valgus posture during provocative and/or complex motor tasks may prove extremely challenging without engaging and effective re-training technologies.

In this review, we will now change focus to biomechanics in those with an ACL injury and post-ACL reconstruction. In most of the developed world, the ruptured ACL is reconstructed using autograft harvested from the semitendinosus alone or in combination with the gracilis (although quadriceps tendon autografts are gaining popularity). The resulting autograft is of excellent size and strength to replace the failed ACL, but the donor sites experience severe and often long-term morbidity, such as variable (and often failed) regeneration of the harvested tendon, proximal migration of the semitendinosus insertion, semitendinosus muscle belly proximal retraction, semitendinosus atrophy and fatty infiltration. These post-ACLR impairments are issues for secondary ACL injury, but also primary hamstring strains as the function of the semitendinosus is impaired, as may be its synergists in their attempt to compensate. Understanding the function of muscles surface electromyography can provide some insight, particularly when combined with computational neuromusculoskeletal modelling (15) which enables subject- and task-specific resolution of the musculoskeletal load-sharing problem. For over two decades, the SENIAM guidelines (16) for electrode placement have served as a standard for surface electromyography. However, due to the morbidity (i.e., atrophy and muscle belly retraction) following ACL reconstruction using semitendinosus tendon, Kositsky et al. have shown surface electrodes applied using the SENIAM guidelines may result in spurious measurement. The authors recommend the application of surface electrodes be done preferably with ultrasound guidance when studying impaired muscle groups. Indeed, the standard locations from SENIAM may fail to be atop the semitendinosus following ACL reconstruction for knee postures ranging from 90° flexion through to full extension. Of note, the spurious EMG conclusions were not made by comparing EMG signals to some empirical standard, but through inference, as the electrodes were not physically atop the impaired semitendinosus. This is relevant to the rehabilitation following ACL reconstruction using the semitendinosus tendon, as EMG recording may be important to retraining recruitment of this impaired structure and is certainly important for computational models of the post-operative muscle and joint function.

The vertical drop jump, in isolation (17), coupled with angular bounding (18) or successive jumps (19) has demonstrated the capacity to reveal deficits in neuromuscular function in those following ACL reconstruction. In the study by **Benoit** and colleagues, they examined healthy athletes performing a vertical drop jump. They suggested that a failed jump landing is initiated prior to ground contact, and that kinematic variables can be used to predict this failure. This raises some interesting motor control questions as in-flight adjustments in body position may not be possible from a mechanics (i.e., Newton's third law and conservation of momentum/energy) or neuromuscular (i.e., reaction times) perspective. The authors have focused their interpretation of the results on the relevance of injury prevention, and indicate programs for reducing injury risk should emphasize controlling the landing preparation, rather than ground contact techniques. This conclusion is consilient with existing literature focusing on trunk control on land as a risk factor for knee injury. A challenge of this study is to understand if the classifications of success and failure (i.e., sticking a single foot landing or stepping with the contralateral leg to maintain balance) during unanticipated tasks have relevance for loading the ACL. Indeed, in many game scenarios, the athletes must make decisions within a minimal time potentially resulting in motor control strategies that expose their ACL (and other soft tissues) to excessive loading. Potentially, sticking the landing increases loading to the ACL, whereas taking steps after landing might lower ACL loading. Alternatively, the need for steps might reflect poor neuromuscular control which might predispose the individual to eventual ACL injury. If so, further efforts should be made to improve the classification accuracy and explore if field-friend technologies (e.g., depth cameras combined with computer vision methods) can identify the kinematics that predicts poor landing.

In addition to the morphological issues documented by Kositsky et al. in the semitendinosus following ACL reconstruction, functional deficits of the knee are common after ACL injury. Stenroth et al. observed that despite having knee flexor and extensor muscle strength deficits post ACL reconstruction, a compensatory increase in the mechanical output of hip extensor muscles assists patients to improve their knee function. The consequence is an overall good-quality movement which might fail to be noticed by visual inspection by clinicians (e.g., rehabilitation specialists). Although explorative in nature, the study highlighted compensatory muscular strategies that are not detectable in lower limb kinematics, which may hide deficits in knee function after an ACL injury. This links well with our introductory remarks that note we must see the fusion of computational modelling with field-friendly (and rugged) instruments to empower clinicians to “see inside” the body and access internal biomechanics. Indeed, as these are the mechanics physically coupled to injury, it behooves us to examine their properties during provocative tasks and during rehabilitation.

Currently, ACL injury prevention programs are defined to provide a set of universal exercises to a team without considering player-specific deficiencies. Usually, the main objective of these programs is to provide sufficient neuromuscular control and strength to the athletes so that they can handle unexpected situations that can cause tissue overload and subsequent rupture (20). The set of proposed exercises usually includes plyometrics, strength training, agility, and balance tasks (21). Their efficacy is assessed through common functional performance tests such as the star excursion balance test, functional hop test, and landing error scoring system (22). The assumption is practicing pre-planned motor skills in a predictable environment will enable the athlete to transfer these competencies toward unpredictable and more complex tasks during play (23). Given the large number of injured athletes worldwide (1), some limitations in this standard approach have been highlighted. The identification of neuromuscular and biomechanical risk factors for ACL injuries in athletes before the assessment of the effectiveness of a prevention programme is lacking in the majority of the studies (24). Then prevention programmes targeted towards the modification of these risk factors are needed. This topic is touched on in the work of **Spolaor and Ciniglio et al.** where a framework for assessing athletes before and after the administration of a preventive protocol is proposed which includes an on-the-field biomechanical assessment of athletes while performing cutting manoeuvres. However, in this work, the personalization of the program based on the biomechanical risk factors identified in the athletes is still lacking.

Our ability to understand the biomechanics of ACL injury prevention, and design nonsurgical interventions that are effective, will greatly benefit from the use of new and innovative integrated technologies. Since habitual motor control strategies are most difficult to modify in day-to-day life, biomechanical measurements should be shifted to the field. In addition to laboratory-based measurement systems for computing movement dynamics, wearable sensors (25) and smartphones videos (26) have recently made them a promising cost-effective alternative, although they do not yet provide precise estimates of mechanical loading. Nonetheless, the use of newer approaches such as artificial intelligence platforms and machine learning (27, 28), virtual reality for injury risk screening (29), return to sports assessment (30), and interactive augmented-reality-based neuromuscular training methods (31) have great potential to lead to a better understanding of the role of biomechanics to stratify ACL injury risk and spur prevention, as well as to improve management and re-injury avoidance of ACL affected people.
